# Factors predicting hospital length-of-stay after radical prostatectomy: a population-based study

**DOI:** 10.1186/1472-6963-13-244

**Published:** 2013-07-02

**Authors:** Maria Kelly, Linda Sharp, Fiona Dwane, Tracy Kelleher, Frances J Drummond, Harry Comber

**Affiliations:** 1 National Cancer Registry, Building 6800, Cork Airport Business Park, Cork, Ireland

**Keywords:** Prostate, Cancer, Length-of-stay, Surgery, Prostatectomy, Service provision

## Abstract

**Background:**

Radical prostatectomy (RP) is a leading treatment option for localised prostate cancer. Although hospital in-patient stays accounts for much of the costs of treatment, little is known about population-level trends in length-of-stay (LOS). We investigated factors predicting hospital LOS and readmissions in men who had RP following prostate cancer.

**Methods:**

Incident prostate cancers (ICD-O3: C61), diagnosed January 2002-December 2008 in men < 70 years, were identified from the Irish Cancer Registry, and linked to public hospital episodes. For those who had RP (ICD-9 CM procedure codes 60.3, 60.4, 60.5, 60.62) the associated hospital episode was identified. LOS was calculated as the number of days from date of admission to date of discharge. Patient-, tumour-, and health service-related factors predicting longer LOS (upper quartile, >9 days) were investigated using logistic regression. Patterns in day-case and in-patient readmissions within 28 days of discharge following RP were explored.

**Results:**

Over the study period 9096 prostate cancers were diagnosed in men under 70, 26.5% of whom had RP by end of follow-up 31/12/2009. Two of eight public hospitals and eight of forty surgeons carried out 50% of all public-service RPs. Median LOS was 8 days (10th-90th percentile = 6-13 days) and fell significantly over time (2002, 9 days; 2008, 7 days; p < 0.001). In adjusted analyses men who were not married (OR = 1.71, 95% CI 1.25-2.34), had co-morbidities (OR = 1.64, 95% CI 1.25-2.16) or stage III-IV cancer (OR = 2.19, 95% CI 1.44-3.34) were significantly more likely to have prolonged LOS. Those treated in higher volume hospitals (annual median >49 RPs) or by higher volume surgeons (annual median >17 RPs) were significantly less likely to have prolonged LOS (OR = 0.34, 95% CI 0.26-0.45; OR = 0.55, 95% CI 0.42-0.71 respectively).

**Conclusion:**

Median LOS after RP decreased between 2002 and 2008 in Ireland but it remains higher than in both England and the US. Although volumes of RPs conducted in Ireland are low, there is considerable variation between hospitals and surgeons. Hospital and surgeon volume were strong predictors of shorter LOS, after adjusting for other variables. These factors point to a need for a comprehensive review of prostate cancer service provision.

## Background

In 2008 prostate cancer was the most common incident cancer in men in the developed regions of the world; in Europe it was estimated to account for 21% of all new cancers diagnosed in men [1]. The increasing incidence observed over the past two decades in many developed countries has been attributed to increased detection as a result of prostate-specific antigen (PSA) testing [2,3]. The Republic of Ireland (ROI), where PSA testing is used extensively in primary care [4], was estimated to have the highest prostate cancer incidence rate in Europe in 2008 [5]. One of the consequences of this extensive testing is that the profile of prostate cancers is changing: the age at diagnosis is falling and higher proportions have localised disease [6].

A range of treatment options are available for prostate cancer, including surgery, radiotherapy and androgen deprivation therapy. Recent European guidelines on prostate cancer treatment recommend radical prostatectomy (RP) for localised disease in patients with a life expectancy of more than 10 years, who accept the risk of treatment-related complications [7]. In the UK RP use has increased rapidly in recent years [8,9], and in the US it is now one of the most common treatment options for localised disease [10,11].

The costs to health services of treating prostate cancer are significant and the initial treatment modality affects both short-term and long-term costs [12]. In 2006, the mean direct cost per patient for initial treatment was over €3500 in the UK and Germany, and over €5,000 in France and Italy [13]. Hospitalisation, both for initial surgery and for any readmissions during the first year of care, account for the largest proportion of these costs [14].

Approaches to RP are evolving rapidly, driven by technological advances; in addition to conventional open surgery, the procedure may be done laparoscopically either by a surgeon alone, or by a surgeon assisted by a robot [15]. It has been suggested that different approaches are associated with variations in post-operative recovery and length-of stay (LOS) [16]. These developments might be expected to have impacted on LOS, but population-level data is limited. It was against this background that we conducted a population-based analysis of time trends in, and factors which predict hospital LOS and readmissions.

## Methods

### Setting

The study setting was Ireland, which has a mixed public-private health care system. All residents are entitled to use the public health system. Public hospitals also offer private health care, and patients can opt to transfer from public to private care at any time. Patients can also choose to be treated in private hospitals. Thus, there are three categories of patients: (1) public patients treated within public hospitals; (2) private patients who pay for treatment within public hospitals, either out-of-pocket or through private health insurance; and (3) private patients who pay for treatment in private hospitals.

### Data sources

The primary data sources for this study were the National Cancer Registry (NCR) and the Hospital In-Patient Enquiry Scheme (HIPE). The NCR records demographic and clinical information for all cancers diagnosed in the population usually resident in Ireland [17]. Treatment received within the first year of diagnosis is also collected. Most (97.5%) registrations are made by tumour registration officers (TROs) who collate and abstract data from various sources - including pathology laboratories, radiotherapy clinics, patient administration systems, and medical records departments - according to internationally agreed guidelines. The remaining registrations are derived from death certificates (2%) and from general practitioners ( < 0.5%). Death certificates are provided by the Central Statistics Office [18]; date and cause of death in cases registered by the NCR are ascertained by linkage to death certificates using probabilistic matching methods. Completeness of case ascertainment is estimated to be in excess of 97% [19].

HIPE is a computer-based information system that collects data on discharges from all acute public hospitals in Ireland [20]. Limited demographic, clinical and administrative data are collected [21]. Data are subject to computer-based edits/checks at data entry and later validation checks [22]. The NCR is provided with all HIPE records which mention cancer or cancer-related procedures such as radiotherapy and chemotherapy. Private hospitals can volunteer to contribute data to HIPE, but coverage is incomplete so, in this study information from HIPE was limited to patients treated in public hospitals (as either public or private patients).

**Figure 1:**
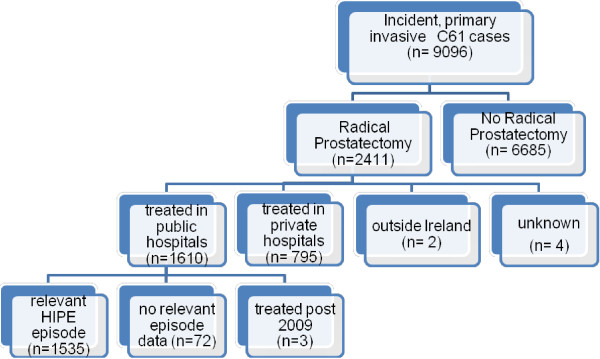
Radical prostatectomy in men aged < 70 years at diagnosis, 2002–2008, dataset overview.

Figure 1 provides an overview of the creation of the analysis dataset. Prostate cancers (ICD10-O3: C61) newly diagnosed between 2002 and 2008 were identified from the NCR. Since RP is rarely conducted in older men [23], the dataset was then limited to men aged < 70 years at diagnosis who had a RP (ICD 9 CM procedure codes 60.3, 60.4, 60.5, 60.62) recorded by the NCR before the end of follow-up on the 31/12/2009. Using probabilistic matching techniques, these prostate cancers were linked to HIPE episodes. We excluded 94 men who had RP in a public hospital but had a previous diagnosis of cancer; 63 of these 94 men were diagnosed in the 12 months prior to the prostate cancer diagnosis. Almost all of the cancers (95%) that occurred in the year prior to the prostate cancer diagnosis were bladder cancer (C67).

Information on patient demographics, clinical and treatment data and provider volume were derived from NCR data. Deprivation was measured using an index created from 2002 census variables which is based on the patient’s area of residence at the time of diagnosis [24]. Information on tumour grade is routinely recorded as part of the cancer registration process using the WHO grading system. Information on Gleason score (GS) recorded in medical records was translated by TROs into WHO grading as follows: GS < 5 = grade 1; GS 5-7 = grade 2; and GS > 7 = grade 3. Stage was defined according to American Joint Committee on Cancer (AJCC) summary staging [25]. For each hospital the annual volume of RPs performed and the median for the entire period was determined. Hospitals were classified as “lower” or “higher” volume by ordering hospitals on the period median volume and splitting so that approximately 50% of patients treated fell into each category. The same approach was used for surgeon volume. Since surgeons can work in more than one hospital and in private and public hospitals, surgeon volume was calculated from NCR data based on all RPs they performed in either public or private hospitals during 2002–2008. “Higher-volume” was defined as >49 RPs per annum for hospitals and >17 RPs per annum for surgeons.

Information on LOS, co-morbidities, admission type (emergency/elective) and discharge status (i.e. whether the patient was “public” or “private” to the consultant at the time of discharge) was derived from HIPE. Each admission (and readmission) was categorised as emergency or elective according to relevant HIPE codes. A co-morbidity count for each patient, based on the Elixhauser index [26] - which was developed to predict a range of outcomes including length-of-stay - was derived from diagnoses recorded on HIPE during the index surgery episode; the prostate cancer diagnosis was disregarded in this calculation.

To determine LOS, HIPE episodes were ordered by date of admission and overlapping episodes combined. The date of first surgery recorded by the NCR was matched to the corresponding HIPE episode to identify the index surgery episode. LOS was defined as the number of days between the date of admission and the date of death or discharge, to either home or another non-acute care facility (e.g. nursing home). For the LOS analysis, cases were excluded if they had: no HIPE episode corresponding to the NCR treatment record; surgery in private hospitals; or surgery after the censoring date (31/12/2009) (Figure 1).

Duration of discharge was calculated as number of days from discharge following the RP to the date of next admission (if any). Patients whose discharge duration was ≤28 days were considered readmissions. This period was chosen for two reasons: firstly, because the 28-day emergency readmission rate is a key hospital performance indicator in some healthcare settings [27]; and secondly because catheters inserted routinely following RP would be expected to have been removed by this time.

### Statistical analysis

Characteristics of patients who had RP and those who did not, and of those who had RP in public and private hospitals, were compared using chi-square tests. Median LOS was determined overall and by year. Trends in LOS by year were examined using the Kruskal-Wallis equality-of-populations rank test [28] and Cuzick’s non-parametric test for trend [29].

Since we are not aware of any national, or internationally, agreed definition of prolonged LOS following RP, in the primary analysis we defined a prolonged hospital stay as a stay of duration greater than the upper-quartile of LOS (>9 days). Multivariable logistic regression was used to identify factors which predicted prolonged LOS. Three types of variables were considered for inclusion in the model: socio-demographic (age, marital status, deprivation index, smoking status at diagnosis, patient status at discharge); clinical (grade, stage, co-morbidity); and care (hospital volume, surgeon volume, year of surgery). Four cases had missing information on marital status and were excluded entirely from the analysis dataset. Cases with missing data on other variables (deprivation index (n = 139), smoking status at diagnosis (n = 328), patient status at discharge (n = 112), grade (n = 43) and stage (n = 1128)) were included as a separate level in the multivariable analysis (see Additional file 1). Variables were included in the multivariable model if they were significant (p < 0.05) on likelihood ratio tests. Model goodness-of-fit was checked using the Hosmer and Lemeshow test [30].

We tested whether the effect of hospital volume on prolonged LOS was modified by surgeon volume by comparing two models: one including an interaction term between hospital and surgeon volume and one without this.

As the median (LOS = 8 days) and 75th percentile (LOS = 9 days) differed by only one day we conducted a sensitivity analyses where we repeated the main analysis using the 90th percentile to define prolonged LOS (>12 days). In a second sensitivity analysis we used aggregated LOS to define prolonged LOS. Aggregated LOS was the sum of LOS for the index RP episode plus all overnight readmissions occurring with 28 days of discharge from the index episode; in this analysis prolonged LOS was defined as the upper-quartile of aggregated LOS for all cases (>10 days).

Readmissions were tabulated by hospital and surgeon volume; these were further categorized into elective and emergency admissions. Reasons for readmission were obtained from HIPE diagnostic codes for the relevant episodes. All analyses were carried out using Stata 11 [31].

## Results

9096 incident prostate cancers were diagnosed in men aged less than 70 years between 2002 and 2008 of whom 26.5% (n = 2411) had a RP by the end of follow-up at 31/12/2009, (Figure 1). Over the entire study period, compared to men who did not have RP, those who did were younger and more frequently had low or intermediate grade disease, were married, resident in less derived areas and had never smoked (Additional file 2). Two thirds (n = 1610) of all RPs were done in public hospitals. Patients treated in a public hospital had a lower mean age, and a greater proportion lived in a deprived area and a higher proportion smoked at diagnosis than those treated in private hospitals (Additional file 2).

The number of hospitals performing RP ranged from a low of 16 in 2002 (8 public, 8 private) to a high of 20 in 2007 (10 public, 10 private). Half of all RPs carried out in public hospitals were done in two facilities; the remainder were carried out in eight institutions. In these eight hospitals the median volume varied from 1 to 43 RPs per year. In total 49 surgeons performed RP over the study period; of these, 9 worked in private hospitals only, 9 worked in both public and private institutions and 31 worked in public hospitals only. The number of surgeons performing RP ranged from 20 in 2002 to 27 in 2008 and was highest (n = 33) in 2007. Eight surgeons performed 50% of all RP done in public hospitals. Median RP volume per surgeon varied from 1 to 17 per year among lower-volume surgeons (annual median ≤ 17 RPs) and from 18 to 36 per year among higher-volume surgeons (annual median >17 RPs).

Just over 95% (n = 1535) of the men who had RP in a public hospital had a relevant HIPE episode (Figure 1). For these men, median LOS was 8 days (10th/90th percentiles = 6/13 days; 25th/75th percentiles = 7/9 days). This decreased significantly from 9 days (10th/90th percentiles = 6/15 days) in 2002 to 7 days (10th/90th percentiles = 6/12) in 2008 (non-parametric test for trend, p < 0.001). Median pre-surgery LOS was 1 day (10th/90th percentiles = 1/2 days) while median post-surgery LOS was 6 days (10th/90th percentiles = 4/11 days).

In multivariable analysis patients who were not married (OR = 1.71, 95% CI 1.25-2.34,), had co-morbidities (OR 1.64, 95% CI 1.25-2.16), or had later stage disease (OR = 2.19, 95% CI 1.44-3.34) were significantly more likely to have a prolonged LOS (upper quartile of LOS >9 days) (Table 1; results for all variables are shown in Additional file 1). Patients treated in higher volume hospitals were significantly less likely to have a prolonged LOS (OR = 0.34, 95% CI 0.26-0.45), as were patients treated by higher volume surgeons (OR = 0.55, 95% CI 0.42-0.71). There was no evidence of an interaction between hospital and surgeon volume (Table 2).

**Table 1:** Factors significantly associated with prolonged LOS in prostate cancer patients undergoing RP in public hospitals^*^

		**LOS > 9 days**			
	**N**^**1**^	**n (%)**^**2**^	**Adjusted OR**^**3**^	**95% CI**	**p-value**^**4**^
**Marital status**					
Married	1268	287 (22.6)	1.00	-	*p < 0.001*
Other	263	87 (33.1)	1.71	1.25-2.34	
**Comorbidity**^**5**^					
None	1127	242 (21.5)	1.00	-	*p < 0.001*
Any	408	133 (32.6)	1.64	1.25-2.16	
**Stage**					
Unknown	1128	255 (22.6)	1.00	-	*p < 0.001*
I & II	285	75 (26.3)	1.38	0.99-1.92	
III & IV	122	45 (37.0)	2.19	1.44-3.34	
**Hospital volume**^**6**^					
Higher (>49)	754	126(16.7)	0.34	0.26-0.45	*p < 0.001*
Lower (≤49)	781	249 (31.9)	1.00	-	
**Surgeon volume**^**7**^					
Higher (>17)	750	161 (21.5)	0.55	0.42-0.71	*p < 0.001*
Lower (≤17)	785	214 (27.3)	1.00	*-*	

^1^total number of men (N), ^2^number and % with prolonged LOS (n, %), ^3^adjusted odds ratios (OR) with 95% confidence intervals (CI) and ^4^p-values, ^5^count of morbidities included in the Elixhauser index on HIPE record relating to the RP episode, 6median number of RPs performed at hospital per year, ^7^median number of RPs performed by surgeon per year in public and private hospitals.

^*^ Variables tested, but not significantly associated with prolonged LOS, and excluded from the final model: age at diagnosis, smoking status at diagnosis, and deprivation index of area of residence, grade/Gleason score and patient status at discharge (public/private to the consultant). See Additional file 1 for the ORs for these variables.

**Table 2:** Risk estimates from alternative models for associations between hospital and surgeon volume and prolonged LOS

**Model**	**Variable (comparison)**	**Adjusted OR**^**1**^	**95% CI**^**2**^	**p-value**
Model 1 (primary analysis): Hospital and surgeon volume both fitted in model (without interaction term)^1^	Hospital volume: (higher vs lower)	0.34	0.26-0.45	
	Surgeon volume: (higher vs lower)	0.55	0.42-0.71	
Model 2: Surgeon volume omitted	Hospital volume: (higher vs lower)	0.37	0.29-0.48	p < 0.001^3^
Model 3: Hospital volume omitted	Surgeon volume: (higher vs lower)	0.63	0.49-0.81	p < 0.001^3^
Model 4: Hospital and surgeon volume fitted with an interaction term^4^	Hospital volume main effect: (higher vs lower)	0.28	0.20-0.40	p = 0.105^**4**^
	Surgeon volume main effect: (higher vs lower)	0.46	0.33-0.64	
	Interaction effect	1.55	0.92-2.65	

^1^Final multivariate model as shown in Table 1, ^2^95% confidence intervals, ^3^p-value for likelihood ratio tests compared to baseline model (model 1), ^4^Model 4 is the baseline model with the addition of an interaction term; the p value is for the interaction term.

One patient died in hospital during the index RP episode and two men died within 28 days of discharge. Of the remainder 55% (n = 854) were readmitted within 28 days, and just under 6% of all readmissions were emergencies (Table 3). In total 47 men were readmitted as emergencies within 28 days. In those men who had a prolonged LOS (>9 days) at the index episode 4.0% were readmitted as emergencies within 28 days (that is 15 emergency readmissions out of 375 men with prolonged LOS at the index episode). In those whose LOS was ≤ 9 days at the index episode 2.8% were readmitted as emergencies within 28 days (that is 32 emergency readmissions out of 1160 men). These two proportions were not significantly different (z = 1.21, p = 0.225).

**Table 3:** Readmissions within 28 days of discharge following RP in public hospitals by provider volume

	**All**	** Hospital volume**^**1**^		** Surgeon volume**^**2**^	
**Volume**	**N (%)**	**Lower**	**Higher**	**Lower**	**Higher**
Number of RPs	1535	781	754	785	750
Number of readmissions^*^	854	344	510	373	481
(% of all RPs)	55.6%	22.4%	33.2%	24.3%	31.3%
**Readmission type**					
Elective - day cases	304	228	76	203	101
(% of all readmissions)	35.6%	26.7%	8.9%	23.8%	11.8%
Elective - overnight	503	99	404	145	358
(% of all readmissions)	58.9%	11.6%	53.6%	17.0%	41.9%
Emergency	47	17	30	25	22
(% of all readmissions)	5.5%	2.0%	3.5%	2.9%	2.6%

^*^excludes those who died at time of index procedure RP (n = 1) or within 28 days of discharge (n = 2),^ 1^higher-volume hospitals are those where >49 RPs were performed per year during 2002–2008, ^2^higher-volume surgeons are those who performed >17 RPs per year during 2002–2008.

Readmissions of any type, and overnight admissions, were more frequent in higher volume hospitals and for higher volume surgeons (Table 3). Catheter removal and urine flow study were the two most common procedures for elective readmission. The most common procedures for emergency readmission were catheter removal, tomography of abdomen, injection of antibiotics or anticoagulants, and endoscopic lavage of blood clots from bladder.

In the sensitivity analysis using the 90th percentile to define prolonged LOS (>12 days), 161 men stayed in hospital for more than 12 days. The factors which significantly predicted longer LOS were the same as in the primary analysis. The effects for all variables were in the same direction as in the primary analysis. The risk estimates for marital status and surgeon volume were slightly further from unity than in the primary analysis (OR not married vs married = 2.13, 95% CI 1.43-3.20; OR higher vs lower surgeon volume = 0.37, 95% CI 0.25-0.54); risk estimates for other variables were very similar to those obtained in the primary analysis (data not shown).

For the sensitivity analysis using aggregated LOS (LOS from the index episode plus any overnight stays within 28 days), median LOS during 2002–2008 was 8 days (10th/90th percentiles = 6/14 days; 25th/75th percentiles = 7/10 days). Median aggregated LOS decreased significantly from 11 days (IQR 9–14) in 2002 to 8 days (IQR 7–9) in 2008, (non-parametric test for trend, p < 0.001). Factors which significantly predicted prolonged aggregated LOS (upper quartile, >10 days) were the same as in the primary analysis (data not shown).

## Discussion

### Strengths and limitations

One of the major strengths of this study is that it is based on high-quality, population-based, cancer registration data (rather than hospital admission data alone), providing confidence that the patients included had prostate cancer. In addition, in our mixed public-private setting, where clinicians frequently work in both sectors, the availability of national cancer registry data made it possible to accurately quantify the entire (i.e. public and private) volume of cases for each surgeon.

Two thirds of men had RP in a public hospital and one third in a private hospital. Just over 4% of publicly-treated patients had no matching HIPE episode. A failure to find a match between cancer registrations and hospital episodes can occur for a number of reasons including: an inability to match records belonging to the same person due to typographical errors or missing information on either system, or no mention of cancer (or cancer-related procedures) on HIPE, in which case the episodes would not be available to the cancer registry. The missing episodes were distributed across hospitals and years.

We excluded 94 men who had another invasive cancer diagnosis (other than a non-melanoma skin cancer) prior to the prostate cancer diagnosis. The majority of these (67%) were diagnosed in the 12 months prior to the prostate cancer diagnosis. The diagnosis and treatment of recent previous cancers are likely to have an impact on the treatment decisions for the prostate cancer and hence may impact on LOS in men undergoing RP. This exclusion means that our results are generalizable to the population of men treated with RP for whom prostate cancer is their first cancer.

### Comparisons in LOS between countries

Our median LOS of 7 days in 2008 is slightly higher than reported in England (median 6 days, based on patients treated in the publically-funded NHS) [9]. Our median post-operative stay was 6 days and is double that reported in the US (post-operative stay 3 days in patients treated through the Medicare health insurance programme) [10]. Our analysis of LOS was confined to men treated in public hospitals only and those treated in public and private hospitals differed in terms of age, smoking status and deprivation category. Although none of these factors were significantly associated with prolonged LOS, it is possible that men treated privately might differ from those treated publicly in other ways that do impact LOS. For example, men treated privately might have fewer or less important co-morbidities or be fit for surgery. Therefore, our results confined to public patients could over-represent sicker or less fit patients or those with more complex disease, therefore driving up median LOS. It is impossible to assess whether this is true, however, since there are no data available on comorbidities, fitness for surgery, or case complexity in private patients.

In the US there has been a concerted effort to reduce post-surgery LOS following RP using collaborative care pathways [32-34]. Further reductions have been driven by increased use of laparoscopic (LRP) and robot assisted RP (RARP) [10]. Unfortunately we did not have information on the type of RP conducted and so could not investigate the impact of these further. However, RARP was not available in public hospitals during the study period. It is not particularly usual for public hospitals in Ireland to document local guidelines for practice, so we would expect that most hospitals would not have established standard post-operative care pathways. Nor has there been any specific national initiatives to reduce LOS during the study period. This, together with differences in casemix, type of procedure performed and public and private healthcare cultures, probably explains the higher LOS in Ireland than in the US.

### Time trends in LOS

There was a modest downward trend in median LOS over the study period, similar to patterns reported elsewhere. In England, for example, Hanchanale et al. [9] reported a decrease in median LOS from 8 days (IQR, 7–10) in 1998 to 6 days (IQR, 5–8) in 2004. The authors reported a modest increase in the use of LRP but this accounted for only 4% of RP cases overall and was considered unlikely to fully explain the downward trend.

A range of national clinical initiatives to improve patient care post-surgery and reduce LOS are only currently being developed in Ireland [35] so cannot have impacted on our study results. However there may have been local initiatives of which we were not aware. Other possible – and plausible – explanations for the observed trend are greater surgical experience resulting from the increased volume of RPs carried out, together with improvements in anaesthetic techniques leading to faster post-operative recovery times [36].

### Factors associated with prolonged LOS

In the adjusted analysis men who were not married, and who had co-morbidities or later stage disease were significantly more likely to have a prolonged hospital stay (>9 days). We found similar associations in patients with colorectal cancer who underwent resection [37]. The observation that married patients have shorter LOS may reflect a lack of social support among unmarried patients [38,39]. Urologists may be reluctant to discharge men who have no identifiable family caregiver. A shortage of step-down beds could exacerbate this problem as patients may occupy hospital beds for longer than required [40]. It might be expected a priori that those with co-morbidities would have longer LOS; these patients may need more investigation and work-up before surgery or a longer recovery time post-surgery. Similarly patients with more advanced disease are likely to need more investigation and work-up before surgery, more extensive surgery and more investigation post-surgery to classify surgical margins and to be assessed for further treatment.

The observed associations between LOS and provider volume have been previously reported [32]. What seems to be uncertain is whether surgeon or hospital volume, or both, is important. Like us, in an analysis of NHS data in England, Hanchanale et al. [9] found both hospital and surgeon volume were inversely associated with LOS. However, Judge et al. [8], using the same source data, found that trust volume alone was important. The need for minimum volume thresholds for urological cancer surgery has been recognised by surgeons [41] and in England the National Institute for Clinical Excellence (NICE) recommends that RP should be provided by specialised teams typically carrying out 50 or more operations per annum [42]. In Ireland only two public hospitals conducted 50 or more RPs per year. Evidence also suggests that experience in a particular mode of RP (i.e. ORP, LRP, RARP) is important for improved patient outcomes [41,43,44].

Although we found that both hospital and surgeon volume were statistically independent predictors of LOS, in practice these are likely to be related; higher volume centres are more likely to have more experienced surgeons who in turn may drive quality improvements or improvements in perioperative care. However, higher-volume centres may also deal with more complex cases which cannot be adjusted for fully, using simple co-morbidity counts [45]. Unfortunately we do not have information on other predictors of case complexity (e.g. frailty), and so we may have underestimated the strength of association between higher volume and lower LOS.

### Readmissions

There was a high rate of overnight elective readmissions following RP and, in further analysis, wide variations between public hospitals were evident. Some private hospitals in Ireland have a stated policy of overnight readmission for catheter removal and it is reasonable to assume that public hospitals with high overnight elective readmission rates have a similar policy. The cost-benefit implications for the health services nationally (and for individual hospitals) of overnight readmission versus elective day-patient care for catheter removal are unclear and warrant further investigation.

Although higher-volume hospitals had a higher overnight elective readmission rate than lower-volume hospitals, the risk of prolonged LOS remained lower in higher-volume hospitals when aggregated LOS (i.e. the sum of LOS for the RP itself and any readmissions within 28 days), rather than the surgery episode LOS, was considered. Although the overall numbers were low, and so need to be interpreted with caution, the data suggested that higher-volume hospitals had more emergency readmissions than lower-volume hospitals; this is probably a reflection of more complex cases being treated at higher-volume hospitals.

### Centralisation

In comparison with the UK and US the total number of RPs undertaken in Ireland is small. In addition, the pool of hospitals and surgeons providing the service is small and this is further complicated by the public/private divide. The fact that 50% of RPs in public hospitals were carried out at two hospitals (and by eight urologists) demonstrates the diffusion of cancer services in Ireland. The National Cancer Control Programme (NCCP) has embarked on a programme of centralization of public cancer services. Greater centralization of prostate cancer services could facilitate the development of a pool of experienced surgeons potentially leading to a more robust service less sensitive to loss of personnel and better equipped to implement technological advances in RP surgery such as LRP and RARP. It would facilitate the implementation of clinical initiatives already shown elsewhere to improve patient and hospital-related outcomes (e.g. standardized critical care pathways) and – as suggested by this study – lead to shorter LOS. It would also enable investigation of other aspects of service provision that could both improve quality-of-care and might reduce costs (e.g. more efficient discharge policies and standardised follow-up care). However, centralisation would be likely to mean that patients would need to travel further to access care causing increased inconvenience and cost and, perhaps, impacting on their satisfaction with care. The relationship between these factors is complex, and a full analysis of cost and benefits is required.

## Conclusion

Median LOS after RP decreased between 2002 and 2008 in Ireland but it remains higher than in both England and the US. Although volumes of RPs conducted in Ireland are low, there is considerable variation between hospitals and surgeons. Hospital and surgeon volume were strong predictors of shorter LOS, after adjusting for other variables. These factors point to the need for a comprehensive review of prostate cancer service provision.

## Competing interests

The NCR holds an unrestricted project grant from Sanofi-aventis to investigate comorbidity in prostate cancer (grant-holders: Sharp L, Comber H).

## Authors’ contributions

MK carried out the analysis and wrote the initial drafts of the manuscript. LS conceived the study, provided statistical support and helped draft the manuscript. FD and TK linked the data. LS, HC and FJD helped with interpretation of the data and results. All authors contributed to the final draft of the manuscript. All authors read and approved the final manuscript.

## Pre-publication history

The pre-publication history for this paper can be accessed here:

http://www.biomedcentral.com/1472-6963/13/244/prepub

## Supplementary Material

Additional file 1 Factors associated with prolonged LOS in men with prostate cancer undergoing radical prostatectomy in public hospitals. Click here for file

Additional file 2 Characteristics of prostate cancer patients in total, and by RP surgery status. Click here for file

## References

[B1] Ferlay J Shin HR Bray F Forman D Mathers C Parkin DM GLOBOCAN 2008, Cancer Incidence and Mortality Worldwide: IARC CancerBase No. 10 [Internet] 2010 Lyon, France: International Agency for Research on Cancer Available from: http://globocan.iarc.fr/

[B2] Potosky AL Miller BA Albertsen PC Kramer BS The role of increasing detection in the rising incidence of prostate cancer JAMA 1995 273 7 548 552 10.1001/jama.1995.03520310046028 7530782

[B3] Brewster D Fraser L Harris V Black R Rising incidence of prostate cancer in Scotland: increased risk or increased detection? BJU Int 2000 85 4 463 473 10.1046/j.1464-410x.2000.00487.x 10691826

[B4] Drummond F Sharp L Comber H Major inter-laboratory variations in PSA testing practices: results from national surveys in Ireland in 2006 and 2007 Ir J Med Sci 2008 177 4 317 323 10.1007/s11845-008-0216-1 18841439

[B5] Ferlay J Parkin D Steliarova-Foucher E Estimates of cancer incidence and mortality in Europe in 2008 Eur J Cancer 2010 46 4 765 781 10.1016/j.ejca.2009.12.014 20116997

[B6] Carsin A Drummond F Black A van Leeuwen P Sharp L Murray L Impact of PSA testing and prostatic biopsy on cancer incidence and mortality: comparative study between the Republic of Ireland and Northern Ireland Cancer Causes & Control 2010 21 9 1523 1531 10.1007/s10552-010-9581-y 20514514

[B7] Heidenreich A Aus G Bolla M Joniau S Matveev VB Schmid HP EAU guidelines on prostate cancer Eur Urol 2008 53 1 68 80 10.1016/j.eururo.2007.09.002 17920184

[B8] Judge A Evans S Gunnell DJ Albertsen PC Verne J Martin RM Patient outcomes and length of hospital stay after radical prostatectomy for prostate cancer: analysis of Hospital Episodes Statistics for England BJU Int 2007 100 5 1040 1049 17784890 10.1111/j.1464-410X.2007.07118.x

[B9] Hanchanale VS McCabe JE Javlé P Radical Prostatectomy Practice in England Urol J 2010 7 4 243 248 21170853

[B10] Hu JC Gu X Lipsitz SR Barry MJ D’Amico AV Weinberg AC Comparative effectiveness of minimally invasive vs open radical prostatectomy JAMA 2009 302 14 1557 10.1001/jama.2009.1451 19826025

[B11] Lowrance WT Elkin EB Jacks LM Yee DS Jang TL Laudone VP Comparative effectiveness of prostate cancer surgical treatments: a population based analysis of postoperative outcomes J Urol 2010 183 4 1366 1372 10.1016/j.juro.2009.12.021 20188381 PMC2866516

[B12] Snyder CF Frick KD Blackford AL Herbert RJ Neville BA Carducci MA How does initial treatment choice affect short‒term and long-term costs for clinically localized prostate cancer? Cancer 2010 116 23 5391 5399 10.1002/cncr.25517 20734396

[B13] Fourcade RO Benedict Á Black LK Stokes ME Alcaraz A Castro R Treatment costs of prostate cancer in the first year after diagnosis: a short‒term cost of illness study for France, Germany, Italy, Spain and the UK BJU Int 2010 105 1 49 56 10.1111/j.1464-410X.2009.08716.x 20132102

[B14] Warren JL Yabroff KR Meekins A Topor M Lamont EB Brown ML Evaluation of trends in the cost of initial cancer treatment J Natl Cancer Inst 2008 100 12 888 10.1093/jnci/djn175 18544740 PMC3298963

[B15] Heer R Raymond I Jackson MJ Soomro NA A critical systematic review of recent clinical trials comparing open retropubic, laparoscopic and robot-assisted laparoscopic radical prostatectomy Rev Recent Clin Trials 2011 6 3 241 249 10.2174/157488711796575513 21682688

[B16] Kommu SS Eden CG Luscombe CJ Golash A Persad RA Initial treatment costs of organ‒confined prostate cancer: a general perspective BJU Int 2011 107 1 1 3 10.1111/j.1464-410X.2010.09885.x 21176067

[B17] The National Cancer Registry, Ireland 2012 Available at: http://www.ncri.ie/ncri/index.shtml. Accessed 6/25/2012

[B18] CSO - Central Statistics Office Ireland 2012 Available at: http://www.cso.ie/en/index.html. Accessed 6/25/2012

[B19] CompletenessQuality.pdf (application/pdf Object) 2012 Available at: http://www.ncri.ie/pubs/pubfiles/CompletenessQuality.pdf. Accessed 6/25/2012

[B20] HIPE 2012 Available at: http://www.esri.ie/health_information/hipe/. Accessed 6/25/2012

[B21] National Centre for Classification in Health Faculty of Health Sciences 2008 6 The University of Sydney, NSW 1825 Australia: National Centre for Classification in Health (Sydney) ICD-10-AM ACHI & ACS

[B22] Wiley M Using HIPE data as a research and planning tool: limitations and opportunities: A Response Ir J Med Sci 2005 174 2 52 57 10.1007/BF03169130 16094912

[B23] de Camargo Cancela M Comber H Sharp L Age remains the major predictor of curative treatment receipt for localised prostate cancer: a population-based study Br J Cancer 2013 Epub ahead of print 10.1038/bjc.2013.268 PMC3708581 23722470

[B24] Kelly A Teljeur C The National Deprivation Index for Health & Health Services Research 2007 Trinity College Dublin: Small Area Health Research Unit (SAHRU)

[B25] Fleming I Cooper J Henson D Hutter R Kennedy B Murphy G American Joint Committee on cancer: AJCC cancer staging manual 1997

[B26] Elixhauser A Steiner C Harris DR Coffey RM Comorbidity measures for use with administrative data Med Care 1998 36 1 8 10.1097/00005650-199801000-00004 9431328

[B27] Indicator portal | The NHS Information Centre 2012 Available at: http://www.ic.nhs.uk/indicatorportal. Accessed 6/25/2012

[B28] Kruskal WH Wallis WA Use of ranks in one-criterion variance analysis J Am Stat Assoc 1957 47 583 621

[B29] Cuzick J A Wilcoxon-type test for trend Stat Med 1985 4 4 543 547 10.1002/sim.4780040416 4089356

[B30] Hosmer DW Hosmer T Le Cessie S Lemeshow S A comparison of goodness-of-fit tests for the logistic regression model Stat Med 1997 16 9 965 980 10.1002/(SICI)1097-0258(19970515)16:9<965::AID-SIM509>3.0.CO;2-O 9160492

[B31] StataCorp Stata Statistical Software: Release 11 2009 College Station, TX: StataCorp LP

[B32] Impact of surgeon and hospital volume on outcomes of radical prostatectomy. Urologic Oncology: Seminars and Original Investigations Vol. 28. No. 3 2010 Elsevier 10.1016/j.urolonc.2009.03.001 19395287

[B33] Chang SS Cole E Smith JA Jr Baumgartner R Wells N Cookson MS Safely reducing length of stay after open radical retropubic prostatectomy under the guidance of a clinical care pathway Cancer 2005 104 4 747 751 10.1002/cncr.21233 15999365

[B34] Hsu YC Tsui KH Chen CL Lee SH Wu YS Chang PL Web-based Clinical Pathway for Reducing Practice Variations in Radical Prostatectomy Chang Gung Med J 2008 31 6 567 575 19241896

[B35] HSE.ie - Health Service Executive Website - Elective Surgery Programme (ESP) 2012 Available at: http://www.hse.ie/eng/about/Who/clinical/natclinprog/elesurgeryprog.html. Accessed 6/25/2012

[B36] Malhorta V Anesthesia considerations radical prostatectomy Conferencias Magistrales 2006 29 s1

[B37] Kelly M Sharp L Dwane F Kelleher T Comber H Factors predicting hospital length-of-stay and readmission after colorectal resection: a population-based study of elective and emergency admissions BMC Health Serv Res 2012 12 1 77 10.1186/1472-6963-12-77 22448728 PMC3341181

[B38] Downing A Lansdown M West RM Thomas JD Lawrence G Forman D Changes in and predictors of length of stay in hospital after surgery for breast cancer between 1997/98 and 2004/05 in two regions of England: a population-based study BMC Health Serv Res 2009 9 202 10.1186/1472-6963-9-202 19900265 PMC2777882

[B39] Pruthi RS Lentz AC Sand M Kouba E Wallen EM Impact of marital status in patients undergoing radical cystectomy for bladder cancer World J Urol 2009 27 4 573 576 10.1007/s00345-009-0380-6 19219612

[B40] Grocott M Browne J Van der Meulen J Matejowsky C Mutch M Hamilton M The Postoperative Morbidity Survey was validated and used to describe morbidity after major surgery J Clin Epidemiol 2007 60 9 919 928 10.1016/j.jclinepi.2006.12.003 17689808

[B41] Nuttall MC van der Meulen J McIntosh G Gillatt D Emberton M Threshold volumes for urological cancer surgery: a survey of UK urologists BJU Int 2004 94 7 1010 1013 10.1111/j.1464-410X.2004.05095.x 15541118

[B42] Urological_Manual.pdf (application/pdf Object) 2012 Available at: http://www.nice.org.uk/nicemedia/pdf/Urological_Manual.pdf. Accessed 6/25/2012

[B43] Djavan B Eckersberger E Finkelstein J Sadri H Farr A Apolikhin O Oncologic, functional, and cost analysis of open, laparoscopic, and robotic radical prostatectomy Eur Urol Suppl 2010 9 3 371 378 10.1016/j.eursup.2010.02.009

[B44] Finkelstein J Eckersberger E Sadri H Taneja SS Lepor H Djavan B Open versus laparoscopic versus robot-assisted laparoscopic prostatectomy: the European and US experience Rev Urol 2010 12 1 35 20428292 PMC2859140

[B45] Urbanek C Turpen R Rosser CJ Radical prostatectomy: Hospital volumes and surgical volumes–does practice make perfect? BMC surgery 2009 9 1 10 10.1186/1471-2482-9-10 19500401 PMC2701919

