# Supplementary material for: Factors predicting hospital length-of-stay after radical prostatectomy: a population-based study
Source: BMC Health Serv Res. 2013 Jul 2;13:244. doi: 10.1186/1472-6963-13-244 (PMC3750445; doi:10.1186/1472-6963-13-244)
Supplement: Additional file 1 — Factors associated with prolonged LOS in men with prostate cancer undergoing radical prostatectomy in public hospitals. [file 1472-6963-13-244-S1.pdf]

Additional file 1: Factors associated with prolonged LOS in men with prostate cancer undergoing radical prostatectomy in public hospitals. Total number of subjects, median, 10th and 90th percentiles, number and percent with prolonged LOS (n, %), crude and adjusted odds ratios (OR) with 95% confidence intervals (CI) and p-values

	LOS			LOS > 9 days (75th percentile)					
	**N**	**[1]M**	**[2]10th-90th**	**n (%)**	**[3]OR**	**95% CI**	**[4]OR**	**95% CI**	**[5]p-value**
**Age at diagnosis**									
<55	353	7	6 - 12	76 (21.5)	0.86	0.62-1.60	-	-	
55-59	460	8	6 - 12	111 (24.1)	1.00	-	-	-	
60-64	455	8	6 - 13	118 (25.9)	1.10	0.82-1.48	-	-	
65-69	267	8	6 - 15	70 (26.2)	1.11	0.79-1.58	-	-	
**Marital status**									
Married	1268	8	6 - 12	287 (22.6)	1.00	-	1.00	-	*p<0.001*
Other	263	8	6 - 15	87 (33.1)	1.69	1.27-2.25	1.71	1.25-2.34	
**Smoking Status**									
Ever	521	8	6 - 13	122 (23.4)	0.87	0.67-1.14	-	-	
Never	686	8	6 - 13	178 (25.9)	1.0	-	-	-	
Unknown	328	8	6 - 12	75 (22.8)	0.85	0.62-1.15	-	-	
**[6]Deprivation Index**									
1 (least deprived)	400	8	6 - 11	82 (20.5)	1.00	-	-	-	
2	193	8	6 - 12	41 (21.2)	1.05	0.69-1.59	-	-	
3	197	8	6 - 13	55 (27.9)	1.50	1.01-2.23	-	-	
4	246	8	6 - 14	61 (24.8)	1.28	0.88-1.87	-	-	
5 (most deprived)	360	8	6 - 13	99 (27.5)	1.47	1.05-2.06	-	-	
Unknown	139	8	6 - 15	37 (26.6)	1.41	0.90-2.20	-	-	
**[7]Comorbidity**									
None	1127	8	6 - 12	242 (21.5)	1.00	-	1.00	-	*p<0.001*
Any	408	8	6 - 15	133 (32.6)	1.77	1.38-2.27	1.64	1.25-2.16	

Additional file 1 continued,

	LOS			LOS >9 days (75th percentile)					
	n=1535	[1]M	[2]10th-90th	n=375 (%)	[3]OR	95% CI	[4]OR	95% CI	[5]p-value
**Grade/Gleason Score**									
Low/intermed (GS<=7)	1345	8	6 - 13	321 (23.9)	1.00	-	-	-	
High (GS>7)	147	8	6 - 14	39 (26.5)	1.15	0.78-1.70	-	-	
Unknown	43	8	6 - 16	15 (34.9)	1.71	0.90-3.24	-	-	
**Stage**									
Unknown	1128	8	6 - 12	255 (22.6)	1.00	-	1.00	-	*p<0.001*
I & II	285	8	6 - 16	75 (26.3)	1.22	0.91-1.65	1.38	0.99-1.92	
III & IV	122	8.5	6 - 14	45 (37.0)	2.00	1.35-2.96	2.19	1.44-3.34	
**Patient status**									
Public	618	8	6 - 13	145 (23.5)	1.00	-	-	-	
Private	805	7	6 - 12	176 (21.9)	0.91	0.71-1.17	-	-	
Unknown	112	9	6 - 15	54 (48.2)	3.03	2.01-4.60	-	-	
**[8]Hospital volume**									
Higher (>49)	754	7	6 - 11	126(16.7)	0.43	0.34-0.55	0.34	0.26-0.45	*p<0.001*
Lower (<=49)	781	8	6 - 15	249 (31.9)	1.00	-	1.00	-	
**[9]Surgeon volume**									
Higher (>17)	750	8	6 - 11	161 (21.5)	0.73	0.58-0.92	0.55	0.42-0.71	*p<0.001*
Lower (<=17)	785	8	6 - 14	214 (27.3)	1.00	-	1.00	-	

[1]median, [2]10th and 90th percentiles, [3]unadjusted odds ratio, [4]adjusted odds ratio for variables shown; model also adjusted for year of surgery, [5]global p-values from likelihood ratio tests, [6]SAHRU 2002 index, [7]count of morbidities included in the Elixhauser index on HIPE record of RP episode, [8]median number of RPs performed at hospital per year, [9]median number of RP performed by surgeon per year in public and private hospitals
