# Supplementary material for: Factors predicting hospital length-of-stay after radical prostatectomy: a population-based study
Source: BMC Health Serv Res. 2013 Jul 2;13:244. doi: 10.1186/1472-6963-13-244 (PMC3750445; doi:10.1186/1472-6963-13-244)
Supplement: Additional file 2 — Characteristics of prostate cancer patients in total, and by RP surgery status. [file 1472-6963-13-244-S2.pdf]

Additional file 2: Characteristics of prostate cancer patients in total, and by RP surgery status

	All prostate (n=9096) (%)	RP - yes (n=2411) (%)	RP - no (n=6685) (%)	X^2 Test	All RPs[1] (n=2411) (%)	Public hosp (n=1610) (%)	Private hosp (n=795) (%)	X^2 Test
**Age at diagnosis**								
<55	1085 (11.9)	513 (21.3)	572(8.6)	687.4, p <0.001	513 (21.3)	370 (23.0)	143 (18.0)	13.3, p =0.004
55-59	1865 (20.5)	723 (30.0)	1142 (17.1)		723 (30.0)	481 (29.9)	240 (30.2)	
60-64	2727 (30.0)	718 (29.8)	2009 (30.1)		718 (29.8)	481 (29.9)	235 (29.6)	
65-69	3419 (37.6)	457 (18.9)	2962 (44.3)		457 (18.9)	278 (17.3)	177 (22.3)	
**Marital status**								
Married	6706 (73.7)	2006 (83.2)	4700 (70.3)	154.0, p<0.001	2006 (82.2)	1334 (82.9)	667 (83.9)	1.67, p=0.795
Other	2312 (25.4)	397 (16.5)	1915(28.6)		397 (16.5)	272 (16.9)	124 (15.6)	
Unknown	78 (0.9)	8 (0.3)	70 (1.0)		8 (0.3)	4 (0.2)	4 (0.5)	
**Deprivation index[2]**								
1 (least)	2051 (22.5)	633 (26.3)	1418 (21.2)	42.0 p< 0.001	633 (26.3)	417 (25.9)	216 (27.2)	28.7, p = 0.001
2	1203 (13.2)	319 (13.2)	884 (13.20		319 (13.2)	203 (12.6)	115 (14.5)	
3	1190 (13.1)	320 (13.3)	870 (13.0)		320 (13.3)	207 (12.9)	111 (14.0)	
4	1483 (16.3)	408 (16.9)	1075 (16.1)		408 (16.9)	257 (16.0)	149(18.7)	
5 (most)	2227 (24.5)	498 (20.7)	1729 (25.9)		498 (20.7)	377 (23.4)	121 (15.2)	
Unknown	942 (10.4)	233 (9.7)	709 (10.6)		233 (9.7)	149 (9.2)	83 (10.4)	
**Smoking status**								
Ever	2551 (28.0)	741 (30.7)	1810 (27.1)	306.0, p< 0.001	741 (30.8)	545 (33.8)	196 (24.6)	61.2, p< 0.001
Never	2828 (31.1)	1025 (42.5)	1803 (27.0)		1020 (42.4)	712 (44.2)	308 (38.7)	
Unknown	3717 (40.9)	645 (26.7)	3072(45.9)		644 (26.8)	353 (21.9)	291 (36.6)	
**Grade/Gleason score**								
Low/intermed (GS<= 7)	6799 (74.7)	2088 (86.6)	4711 (70.5)	265.3, p<0.001	2088 (86.6)	1408 (87.4)	678 (85.3)	2.4, p=0.305
High (GS>7)	1391 (15.3)	243 (10.1)	1148 (17.2)		243 (10.1)	151 (9.4)	90 (11.3)	
Unknown	906 (10.0)	80 (3.3)	826 (12.4)		80 (3.3)	51 (3.2)	27 (3.4)	
**Stage**								
I & II	1617 (17.8)	449 (18.6)	1168 (17.5)	43.4, p<0.001	448 (18.6)	304 (18.9)	144 (18.1)	1.74, p=0.419
III & IV	1038 (11.4)	187 (7.8)	851 (12.7)		185 (7.7)	131 (8.1)	54 (6.8)	
Unknown	6441 (70.8)	1775 (73.6)	4666 (69.8)		1772 (73.7)	1177 (73.0)	597 (75.1)	

[1]6 patients were treated outside Ireland or in 'unknown' hospitals, [2]SAHRU 2002 index of deprivation
